# Deep‐pelagic fishes: Demographic instability in a stable environment

**DOI:** 10.1002/ece3.11267

**Published:** 2024-04-18

**Authors:** Max D. Weber, Travis M. Richards, Tracey T. Sutton, Joshua E. Carter, Ron I. Eytan

**Affiliations:** ^1^ Texas A&M University at Galveston Galveston Texas USA; ^2^ Nova Southeastern University Dania Beach Florida USA; ^3^ Department of Biological Sciences Louisiana State University Baton Rouge Louisiana USA

**Keywords:** bathypelagic, climate change, mesopelagic, population ecology, population genetics

## Abstract

Demographic histories are frequently a product of the environment, as populations expand or contract in response to major environmental changes, often driven by changes in climate. Meso‐ and bathy‐pelagic fishes inhabit some of the most temporally and spatially stable habitats on the planet. The stability of the deep‐pelagic could make deep‐pelagic fishes resistant to the demographic instability commonly reported in fish species inhabiting other marine habitats, however the demographic histories of deep‐pelagic fishes are unknown. We reconstructed the historical demography of 11 species of deep‐pelagic fishes using mitochondrial and nuclear DNA sequence data. We uncovered widespread evidence of population expansions in our study species, a counterintuitive result based on the nature of deep‐pelagic ecosystems. Frequency‐based methods detected potential demographic changes in nine species of fishes, while extended Bayesian skyline plots identified population expansions in four species. These results suggest that despite the relatively stable nature of the deep‐pelagic environment, the fishes that reside here have likely been impacted by past changes in climate. Further investigation is necessary to better understand how deep‐pelagic fishes, by far Earth's most abundant vertebrates, will respond to future climatic changes.

## Graphical abstract

In this study, we tested the hypothesis that a stable environment should lead to stable demographic histories for the species occupying that environment. We used molecular data to investigate the demographic histories of 11 species of fishes residing in the deep‐pelagic, one of the most stable environments on the planet. Contrary to expectations, we inferred multiple changes in effective population size in the study species.

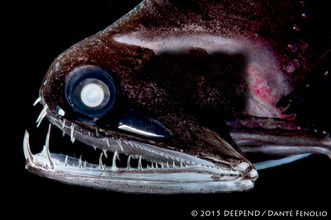

## 1 INTRODUCTION

The demographic history of a species is strongly influenced by the environment it inhabits (Alheit & Hagen, 1997; Avise, 2000; Grant, 2015). Major changes in the environment can alter the distribution and size of suitable habitat for a species, reducing or increasing the species' range. Population sizes expand or contract in response to these fluctuations in habitat suitability (Avise, 2000; Nye et al., 2009). Evidence for the environment's control over population dynamics can be seen across taxonomic groups in terrestrial and marine habitats around the world (Almada et al., 2012; Eytan & Hellberg, 2010; Grant, 2015; Robalo et al., 2012).

Given that changes in environmental conditions strongly influence population size, species inhabiting unstable environments should be characterized by unstable population sizes. On the other hand, species inhabiting temporally stable environments should be less susceptible to frequent population expansions or contractions due to global or regional climatic events. Studies have supported this notion, finding genetic diversity to be greater in species inhabiting more stable environments than closely related species in environments more subject to change (Carnaval et al., 2009; Gugger et al., 2013).

The open ocean mesopelagic (200–1000 m depth) and bathypelagic (1000 m to approximately 100 m above the sea floor) domains (deep‐pelagic, cumulatively) are arguably the most temporally and spatially stable environments on the planet. In terms of physical characteristics like temperature, there is a strong latitudinal homogeneity in the environment that increases with depth (Robison, 2009). In comparison to shallower habitats, temperature change occurs slowly and the magnitude of change is less (Abraham et al., 2013; Clark et al., 2006, 2009; Levitus et al., 2012; Mora et al., 2013; Robison, 2009). Regarding age, the deep‐pelagic milieu has existed longer than continents; while the latter have shifted position, uplifted, submerged, and fractionated, the former have remained relatively unchanged, inter‐ocean connectivity notwithstanding. Based on the age and stability of the environment, as well as our current understanding of the manner in which habitat influences demography, the population sizes of the fishes inhabiting the deep‐pelagic would be expected to be stable over time.

If the demographic histories of deep‐pelagic fishes include population size fluctuations, it is difficult to predict which physical factors could drive this instability. Molecular analyses are frequently used to reconstruct and infer historical demography, however molecular investigations into the historical demography of deep‐sea organisms are few and have focused on deep‐benthic species (Etter et al., 2005; Sakuma et al., 2014; Varela et al., 2012). The deep‐benthic environment, benthic habitat found below 200 m depth, is much more heterogeneous than the deep‐pelagic and likely under differing environmental pressures (Sutton et al., 2017; Thurber et al., 2014; Watling et al., 2013). Recent publications based on the fossil record have reported local changes in deep‐pelagic fish abundance and community composition (Lin et al., 2023; Salvatteci et al., 2022). These observations could be indicative of range changes and fluctuations in population size. Correlations between climate have been made to these findings, but the precise mechanism driving the phenomenon is not yet known.

The physical factors influencing the historical demography of marine fishes inhabiting the less homogenous coastal and epipelagic (upper 200 m of the ocean water column) zones are better understood. Studies have consistently shown population size changes that correspond with major changes in these environments that would not be expected in the more homogenous, ‘sheltered’ deep‐pelagic domain. A plurality of these studies indicates widespread population expansions in shallow‐dwelling fishes following the last glacial maximum (Avise, 2000; Eytan & Hellberg, 2010; Grant, 2015; Robalo et al., 2012). Two factors are frequently cited to explain increases in geographic range and a corresponding increase in population size: an increase in global sea‐surface temperatures and sea‐level rise that dramatically increased shelf habitats (Avise, 2000; Eytan & Hellberg, 2010; Grant, 2015) During this period of great change for shallow marine habitats, the deep‐pelagic experienced far smaller changes in temperature, and the amount of deep‐pelagic habitat would have increased negligibly (Abraham et al., 2013; Clark et al., 2006, 2009; Levitus et al., 2012; Robison, 2009).

If the demographic histories of deep‐pelagic fishes reveal large‐scale population fluctuations, it is possible that physical conditions external to the deep‐pelagic domain drive these dynamics. One habit shared by many deep‐pelagic fishes could be responsible, diel vertical migration.

Most mesopelagic fish species perform diel vertical migrations, a nocturnal migration to the shallower and more variable epipelagic waters and a diurnal return to mesopelagic depths (Barham, 1966; Sutton, 2013). Vertical migrations by bathypelagic fishes, while not unknown are much less common (Cook et al., 2013). An analysis of the distribution of mesopelagic fishes found that the ranges of vertically migrating species were more likely to change in response to large‐scale changes in climate than the ranges of species that do not vertically migrate (Hsieh et al., 2009). This could be a result of the greater influence of atmospheric heating on the upper ocean than deep waters. If the changes in surface waters are no longer physiologically tolerable to vertically migrating fishes, then these species would no longer persist in their former range. If vertical migratory behavior alone drives demography in deep‐pelagic fishes, vertical migrators should be characterized by population expansions and/or contractions, while the population sizes of non‐vertical migrators should be relatively stable over time.

Given the current lack of knowledge regarding deep‐pelagic fish historical demography, we sought to investigate the demographic history of 11 species inhabiting this environment, using two sets of molecular based analyses: frequency‐based tests and gene tree‐based analyses. These tests can infer demographic events such as population expansion and complement one another. Knowledge of the demographic history of deep‐pelagic fishes serves two key purposes. First, it will provide insight into the ecological processes driving population dynamics in the world's largest and most environmentally stable ecosystem. These insights provide the basis for an ideal case study to test the hypothesis that a stable environment should in turn lead to stable demographic histories. Second, understanding how deep‐pelagic fishes responded to past climatic events will allow us to make predictions about how they will respond to future changes in climate.

## 2 METHODS

### 2.1 Sampling and sequence generation

**FIGURE 1:**
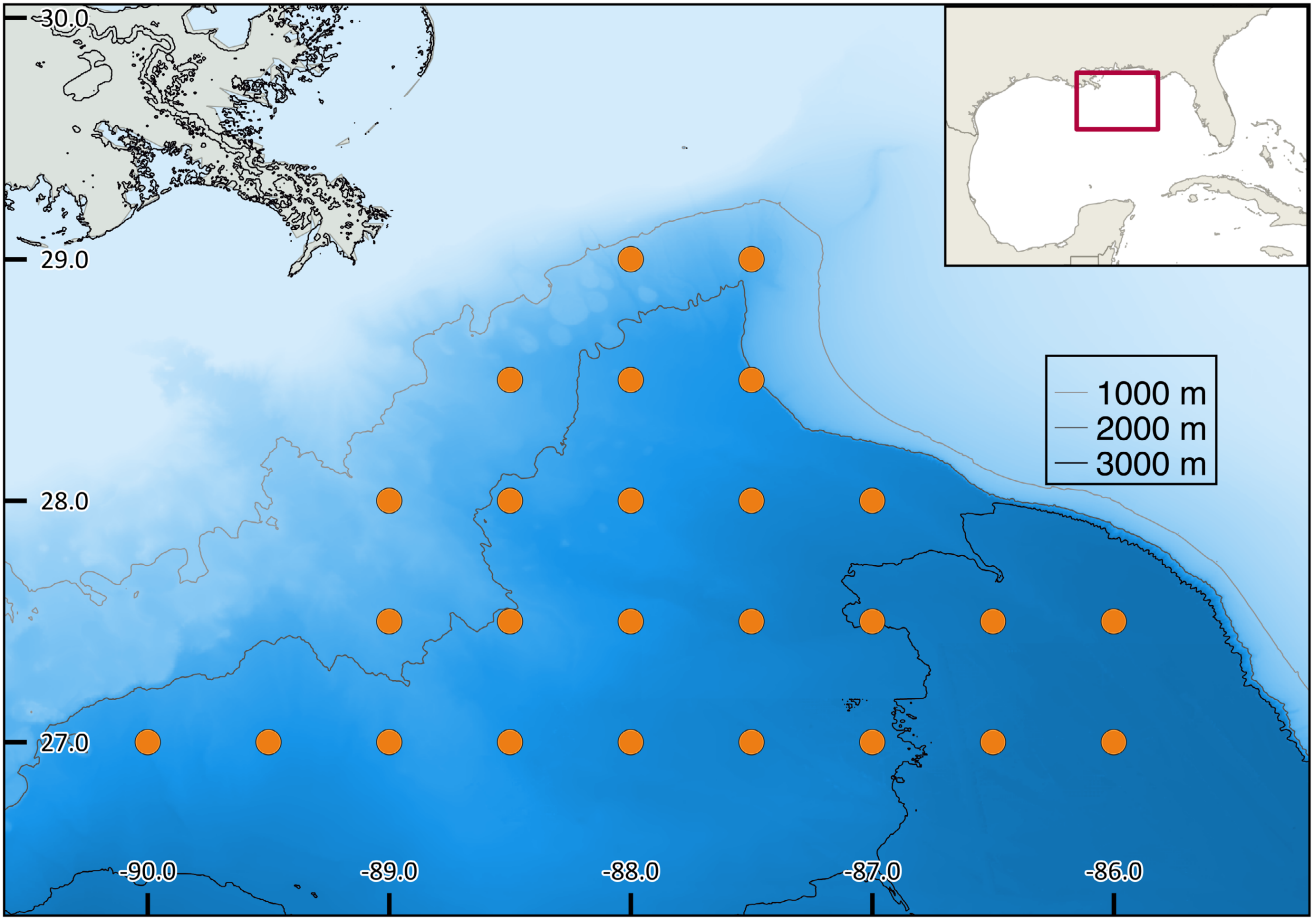
Map of the sampling locations. Station locations are indicated by the black dots, and depth is indicated according to color.

We selected 11 deep‐pelagic species that span phylogenetic lineages, life histories, and vertical migration behavior (see Table 1). Samples were obtained by trawling with a MOCNESS (Multiple Opening and Closing Net and Environmental Sensing System) in discrete depth zones from the surface to 1500 m depth in the northern Gulf of Mexico (GOM) (see Figure 1 for sampling locations). Upon collection and identification of vouchers at sea, a ~1 cm strip of lateral muscle tissue was preserved in 95% non‐denatured ethanol, stored at −20°C while at sea, and moved to long‐term storage at −80°C when back on land. Voucher specimens are housed in the Ocean Ecology Lab at Nova Southeastern University pending accession into a permanently curated fish collection.

**TABLE 1:** Life history traits and taxonomic data for the study species.

Species	Family	Order	Diel vertical migrators	Upper depth of occurrence	Lower depth of occurrence	Total depth range	References
*Bathophilus pawneei*	Stomiidae	Stomiiformes	Yes	0	1500	1500	McEachran and Fechhelm (1998) and Sutton and Hopkins (1996)
*Chauliodus sloani*	Stomiidae	Stomiiformes	Yes	0	1800	1800	McEachran and Fechhelm (1998), Clarke (1983) and Sutton and Hopkins (1996)
*Cyclothone alba*	Gonostomatidae	Stomiiformes	No	300	600	300	McEachran and Fechhelm (1998) and Miya and Nemoto (1986)
*Cyclothone pseudopallida*	Gonostomatidae	Stomiiformes	No	300	900	600	McEachran and Fechhelm (1998) and Miya and Nemoto (1986)
*Diplospinus multistriatus*	Gempylidae	Perciformes	Yes	100	1000	900	McEachran and Fechhelm (1998) and Clarke and Wagner (1976)
*Ditropichthys storeri*	Cetomimidae	Stephanoberyciformes	No	650	2150	1500	McEachran and Fechhelm (1998) and Paxton (1989)
*Photostomias guernei*	Stomiidae	Stomiiformes	Yes	15	800	785	Clarke (1983) and Sutton and Hopkins (1996)
*Scopelogadus mizolepis*	Melamphaidae	Stephanoberyciformes	Yes	100	1000	900	McEachran and Fechhelm (1998), Clarke (1983) and Clarke and Wagner (1976)
*Sigmops elongatus*	Gonostomatidae	Stomiiformes	Yes	50	1200	1150	McEachran and Fechhelm (1998) and Lancraft et al. (1988)
*Sternoptyx pseudobscura*	Sternoptychidae	Stomiiformes	No	800	1500	700	McEachran and Fechhelm (1998)
*Stomias affinis*	Stomiidae	Stomiiformes	Yes	50	850	800	McEachran and Fechhelm (1998) and Sutton and Hopkins (1996)

DNA was extracted from tissues using a Qiagen DNEasy Blood & Tissue extraction kit (Germantown, MD, USA). We generated DNA sequence data from the mitochondrial gene cytochrome oxidase I (COI) as well as three nuclear DNA exons (PLAG, ENC, and MYH). PCR was performed using Promega GoTAQ (Madison, Wisconsin, USA) (see Table A2 for primers used). Following amplification, all PCR products were cleaned using a standard PEG protocol (Glenn, 2019). Amplicons were Sanger‐sequenced on an ABI 3730 capillary sequencer at Yale Keck Biotechnology Resource Laboratory. Sequences were cleaned and edited in Sequencher v5.1. Nuclear markers were phased using Phase v2.1 to resolve heterozygous sites (Stephens & Scheet, 2001, 2005). The sequences were then aligned using MAFFT in Geneiousv9.1.8 (Kearse et al., 2012).

### 2.2 Frequency‐based analyses

We calculated Tajima's *D*, Fu's *F*_s_, and *R* ^2^ for each marker (Fu, 1997; Ramos‐Onsins & Rozas, 2002; Tajima, 1989). Comparisons of the statistical power of frequency‐based tests have shown that *F*_s_ and *R* ^2^ are the most capable of detecting population growth (Ramos‐Onsins & Rozas, 2002). They complement one another as well, with *F*_s_ excelling at population growth detection in large sample sizes, while *R* ^2^ performs better with small sample sizes. A significant and large negative *F*_s_ value suggests population growth, while a significant and small positive *R* ^2^ value indicates population growth. Tajimas's *D* points to population growth and/or a selective sweep when significant and negative.

All of the frequency‐based tests were performed in DNAsp v6 (Rozas et al., 2017). Ambiguity codes were replaced with Ns to allow for calculation in DNAsp. Significance of Tajima's *D* results are determined by the test itself. The significance of all three tests was also determined using coalescent simulations with 1000 replicates implemented in DNAsp.

### 2.3 Gene tree‐based analysis

The second set of tests makes use of the topologies and branch lengths of gene trees to infer changes in population size over time using the coalescent. We performed these analyses in BEAST v2.4.7 (Bouckaert et al., 2014) to generate extended Bayesian skyline plots (EBSPs). EBSPs utilize coalescent theory and a Markov Chain Monte Carlo Algorithm to infer and visualize demographic changes in a dataset. The Bayesian skyline plot is preferable to earlier skyline plot methods as it models both genealogy and demographic history simultaneously, which reduces error rates from uncertainty in estimates of node time (Heled & Drummond, 2008; Ho & Shapiro, 2011).

Nuclear and mitochondrial genes were included in the analysis for each species. The chain length was set to 50,000,000 sampling every 1000. COI rates were fixed, while the nuclear rates were allowed to vary. The partitioning scheme and substitution models were set based on PartitionFinder v2.0 results (Lanfear et al., 2012). A second set of trees was created using the same methodology, with the exception of the selection of substitution models. All partitions were set to the RBS substitution model (Bouckaert et al., 2014). RBS is a reversible‐jump based substitution model for nucleotide data. This substitution model does not require a fixed substitution model to be assigned to each partition at the beginning of the analysis. Instead, it allows five different substitution models to be explored through the run, to find the substitution model with the best fit to the dataset.

After running in BEAST, log files for both sets of trees were inspected using Tracer v 1.7.1 (Rambaut et al., 2014). The most strongly supported EBSP analysis, based on ESS values, was selected and used for the inference of each species' demographic history. The posterior estimate of the number of population size changes provided a test for a rejection of constant population size. A stable demographic history can be rejected in species that do not include a possibility of zero demographic events in this posterior estimate. Finally, the trees files were uploaded to Rstudio v 0.99.484 (Studio 2012). The Rscript “plotEBSP”, provided with the EBSP tutorial (http://www.beast2.org/files/2016/01/ebsp2‐tut.zip), was used to generate and visualize the extended Bayesian skyline plots to understand the nature of these demographic events (Heled, 2010).

We treated each species as a single population for the purpose of these analyses. The accuracy of EBSP results require that the sequences were derived from a single population. The small size of the sampling area (Figure 1) and lack of geographic barriers for dispersal, make this a reasonable assumption. This assumption is supported by genome wide, temporal (multi‐year) analyses of three species within the deep‐pelagic family Myctophidae that reside in the northern Gulf of Mexico (Bernard et al., 2022). Very little instraspecific structure was found, and the authors characterize the GOM lanternfishes as largely panmictic. The samples used in our study were collected alongside those used in the Myctophid research. Given the overlap in habitat, range, and life history traits between lanternfishes and our study species, we believe it is reasonable to assume panmictic populations for our study species within the northern Gulf of Mexico.

### 2.4 Population dynamics and vertical migration

We placed species into two categories; those that had undergone an inferred population size change and those that had not. Species placed into the “inferred population size change” group were categorized as such if the inference was uncovered in both the frequency‐based and gene tree‐based analyses. We further divided species into vertical migrators and non‐vertical migrators. The migration type for each species can be found in Table 1. A chi‐squared test was used to test for a correlation between inferred population size changes and vertical migration.

## 3 RESULTS

### 3.1 Summary

**FIGURE 2:**
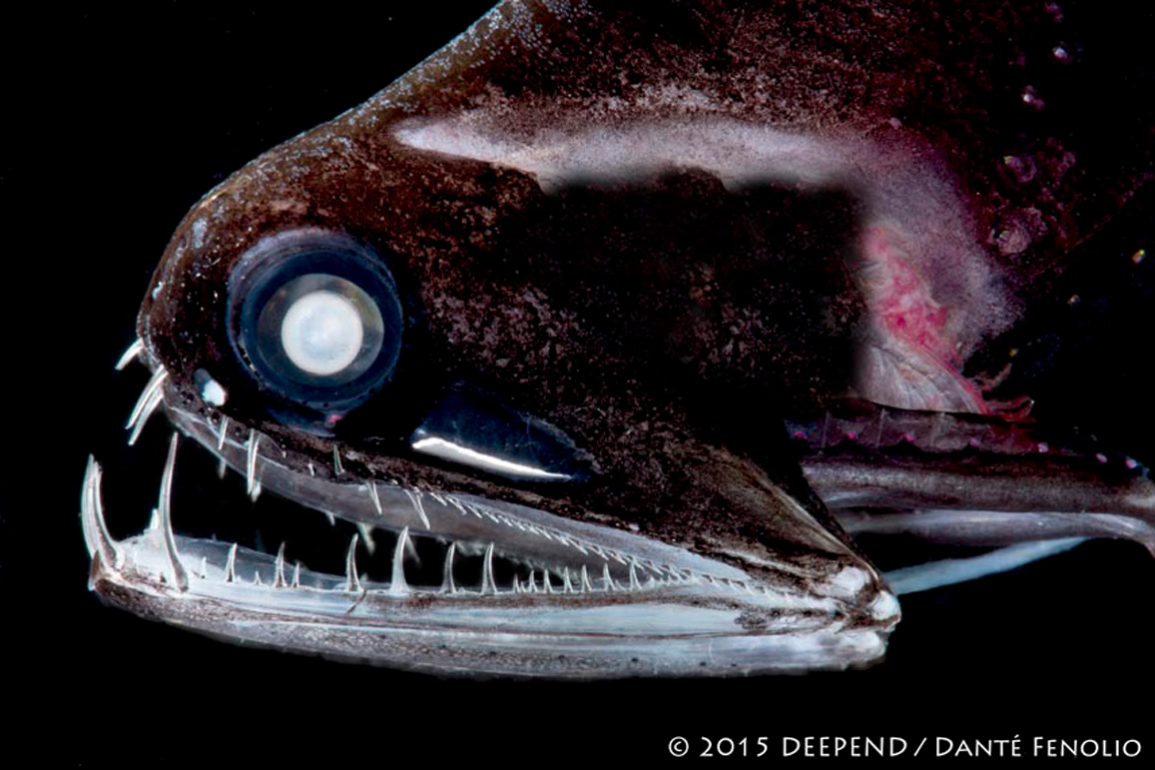
Photo of *Photostomias guernei*. Photo by Dante Fenolio.

The number of sequences generated for each species and marker varied according to sample availability and our ability to achieve amplification. The number of unique sequences obtained for each gene ranged from 10 to 97 (Table 2). The COI dataset included a low of 10 of sequences (*Bathophilus pawnee*i) and a high of 97 sequences (*Chauliodus sloani*). The PLAG dataset included a low of 10 sequences (*B. pawneei*, *Cyclothone pseudopallida*, and *Photostomias guernei*) and a high of 17 sequences (*Sternoptyx pseudobscura*) (see Figure 2 for Photo of *P. guernei*). The ENC dataset included a low of 12 sequences (*Diplospinus multistriatus*) and a high of 16 sequences (*S. pseudobscura*). Finally, the MYH dataset included 11 sequences (*S. pseudobscura*) and 15 sequences (*Stomias affinis*). We used two genes for analysis in nine species, three genes in one species, and four genes in one species (Table 2). All sequences have been deposited in Genbank (Accession numbers listed in Table A1).

**TABLE 2:** Results of frequency‐based statistics analysis.

Species	COI				PLAG				ENC				MYH			
	# Of sequences	Tajima's *D*	*R* ^2^	*F*_s_	# Of sequences	Tajima's *D*	*R* ^2^	*F*_s_	# Of sequences	Tajima's *D*	*R* ^2^	*F*_s_	# Of sequences	Tajima's *D*	*R* ^2^	*F*_s_
*Bathophilus pawneei*	10	1.21	.206	1.761	10	−0.395	.146	−0.07	NA	NA	NA	NA	NA	NA	NA	NA
*Chauliodus Sloani*	97	−2.124	.027	−33.567	NA	NA	NA	NA	19	−2.162	.046	−10.151	NA	NA	NA	NA
*Cyclothone alba*	12	−1.831	.2	−1.008	12	−1.591	.068	−4.89	NA	NA	NA	NA	NA	NA	NA	NA
*Cyclothone pseudopallida*	14	−0.026	.133	−0.68	10	−0.023	.137	0.216	NA	NA	NA	NA	NA	NA	NA	NA
*Diplospinus multistriatus*	12	−1.141	.267	−0.476	12	−0.163	.126	0.2	12	−1.863	.073	−5.836	NA	NA	NA	NA
*Ditropichthys storeri*	11	−0.796	.137	−0.865	10	−2.186	.074	−5.778	NA	NA	NA	NA	NA	NA	NA	NA
*Photostomias guernei*	12	−1.83	.096	−3.216	12	−1.346	.086	−2.582	NA	NA	NA	NA	NA	NA	NA	NA
*Scopelogaus mizolepis*	11	−0.786	.182	−2.995	11	−1.165	.121	−0.097	NA	NA	NA	NA	NA	NA	NA	NA
*Sigmops elongatus*	12	−1.141	.227	−0.476	12	−1.494	.096	−2.383	NA	NA	NA	NA	NA	NA	NA	NA
*Sternoptyx pseudobscura*	13	−1.149	.227	−0.537	17	−1.993	.046	−9.189	16	−2.41	.059	−6.027	15	−1.346	.074	−2.209
*Stomias affinis*	11	−1.673	.07	−8.668	NA	NA	NA	NA	NA	NA	NA	NA	11	−0.477	.115	0.43

*Note*: Tajima's *D* values that were significant based on the two‐tailed test are dark gray. Significant values determined through coalescent simulations are highlighted in light gray.

### 3.2 Frequency‐based analyses

Frequency‐based analyses recovered population expansions in 9 of our 11 sampled species (Table 2). In four species (*C. sloani*, *S. pseudobscura*, *Cyclothone alba*, and *P. guernei*) more than half of the frequency‐based tests for all markers inferred population size changes. Weaker support was present in another five species (*D. multistriatus*, *Ditropichthys storeri*, *Scopelogadus mizolepis*, *Sigmops elongatus*, and *S. affinis*), where less than half of the markers tested produced significant results. No evidence for demographic change was present in *B. pawneei* or *C. pseudopallida*.

### 3.3 Gene tree based analysis

**FIGURE 3:**
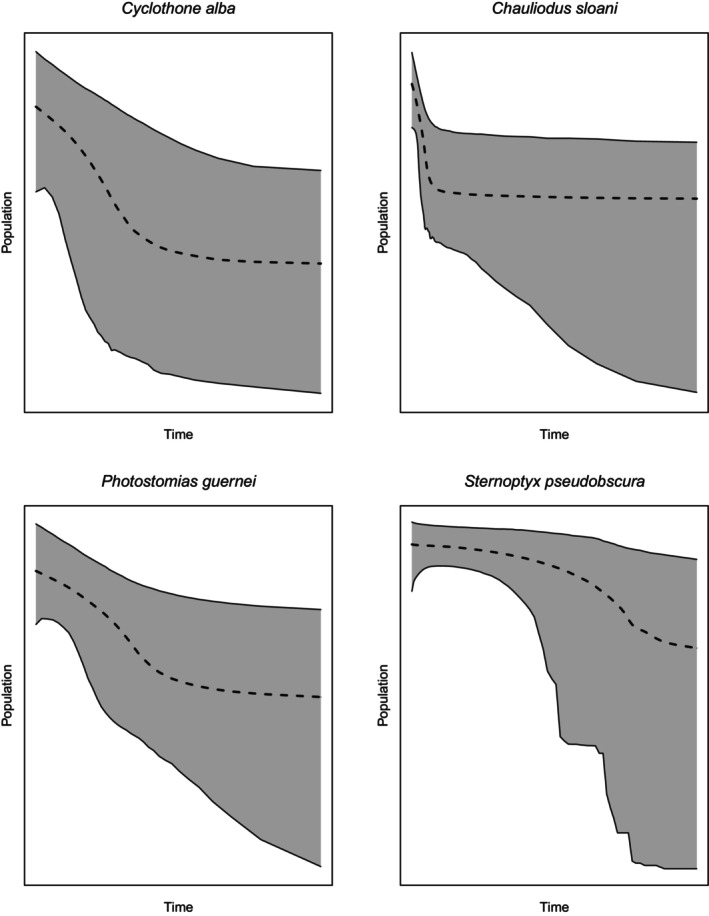
Extended Bayesian skyline plots. The *x*‐axis represents time, with the left side of the axis being the most recent time point. The *y*‐axis represents relative population size on a log scale. The gray area displays the 95% central posterior density.

We were able to reject a stable demographic history in four of the 11 species; *C. alba*, *C. sloani*, *P. guernei*, and *S. pseudobscura*, based on the posterior estimate of the number of population size changes generated in the analyses (Table 3). The estimates suggest a minimum one demographic event and maximum of three demographic events. Population expansions were inferred in every case based on the EBSPs (Figure 3). These four species also shared the strongest evidence for population size changes based on the frequency‐based analyses.

**TABLE 3:** Posterior estimate of population sizes changes.

Study species	Reject constant population	Posterior estimate of population size changes
*Bathophilus pawneii*	No	[0, 3]
*Chauliodus Sloani*	Yes	[1, 3]
*Cyclothone alba*	Yes	[1,3]
*Cyclothone pseudopallida*	No	[0,3]
*Diplospinus multistriatus*	No	[0,3]
*Ditropichthys storeri*	No	[0,3]
*Photostomias guernei*	No	[1,3]
*Scopelogaus mizolepis*	No	[0,3]
*Sigmops elongatus*	No	[0,3]
*Sternoptyx pseudobscura*	Yes	[1,3]
*Stomias affinis*	No	[0,3]

*Note*: These estimates were generated using the gene tree based analyses and provide a test to reject a stable demographic history.

### 3.4 Vertical migration and population dynamics

The chi‐squared test did not provide support for a relationship between vertical migration and population size changes (*p*‐value .8190, Table 4). Of the four species with strong support for population expansions, two (*C. sloani* and *P. guernei*) are vertical migrators while two (*C. alba* and *S. pseudobscura*) do not vertically migrate.

**TABLE 4:** Chi‐squared test for significance of vertical migration on the inference of recent population size changes.

	Pop size change inferred	No pop size change inferred		
Vertical migrator	4	3	Chi‐squared	0.0524
Non vertical migrator	2	2	*p*‐Value	0.819

## 4 DISCUSSION

Historic changes in population size are frequently inferred for marine fishes and attributed to major ecological events and past climatic change (Avise, 2000; Grant, 2015). Previous molecular studies however, have largely focused on marine species inhabiting shallower environments that are more variable over time in terms of physical conditions such as temperature in comparison to the deep‐pelagic. Given the temporal and abiotic stability of the deep‐pelagic environment, population sizes of fishes inhabiting this environment might also be predicted to be stable, so that no effective population size changes would be inferred from genetic examination. Nonetheless, we uncovered multiple lines of evidence suggesting population expansions in four of the 11 study species, while demographic events were inferred in an additional five species using frequency‐based tests.

### 4.1 Interpretation of frequency‐based statistics

Departures from neutrality frequently occur due to past demographic events, however other factors such as selective sweeps and reproductive skew may lead to departures from neutrality as well (Birkner et al., 2013; Eldon et al., 2015; Montano, 2016). This has been shown to be true in several marine fishes (Eldon et al., 2015; Niwa et al., 2016). We sought to avoid such issues by employing a multilocus approach, including both mitochondrial and nuclear genes. Selection could be leading to a positive result on a single gene, however it would be unlikely to be acting on multiple unrelated genes in concert.

### 4.2 Both frequency‐based statistics and EBSPs suggest population expansions

Population expansions were indicated using two different types of analyses that were largely in agreement, however the EBSPs suggested fewer instances of demographic change. It is not surprising that these tests would not agree in every case. Simulated datasets demonstrate that the EBSP analyses are prone to false negatives when using fewer than eight loci (Heled & Drummond, 2008). Given the number of loci sequenced, it seems likely that our EBSPs were more conservative in their inference of population expansions than the frequency‐based tests.

The generation of sequence data was complicated due to the evolutionary distance separating these species, over 200 million years in some cases (Near et al., 2012). Finding primer sets that successfully amplified genes in multiple study species was the limiting to factor to the number of sequences included in this investigation. In the future, the use of high‐throughput sequencing would greatly expand the number of genes available for analyses and provide greater resolution to the demographic histories of the fishes inhabiting the deep‐pelagic, likely increasing the number of demographic expansions that are inferred.

### 4.3 Genetic evidence furthers recent findings based on the fossil record

While molecular techniques can infer demographic history directly, analyses of the fossil record can uncover trends in local species abundance and community composition over time, which can be indicative of larger demographic trends. Several recent publications have used the fossil record to recreate prehistoric deep‐pelagic fish communities. Salvatteci et al. (2022) identified fossilized vertebrae to compare the community structure of fishes inhabiting Humboldt Current at two points in time (the last interglacial and the Holocene) (Salvatteci et al., 2022). The study includes two deep‐pelagic species, with one species increasing and the other decreasing in frequency in the Holocene sample. Lin et al. 2023) utilized fossilized otoliths to investigate trends in deep‐pelagic fishes in the Warm Pacific Pool over the last 460 thousand years (Lin et al., 2023). They included species from five major deep‐pelagic families and found temporal changes in the number of otoliths present in the fossil record as well as the community composition. While some species remained well represented throughout the record others fluctuated greatly. These inferred changes in community composition and abundance could be a record of large‐scale fluctuations in range and population sizes in deep‐pelagic fishes. Given our widespread inference of population instability, it seems likely that the fossil‐based analyses are identifying the same phenomenon.

### 4.4 Potential drivers of deep‐pelagic fish population dynamics

Our widespread inference of population expansions in deep‐pelagic fishes was unexpected. Demographic events are typically attributed to major climate changes that alter the environment, in turn increasing or decreasing the amount of optimal habitat for a given species (Avise, 2000; Grant, 2015). Populations expand or contract in response to these alterations in optimal habitat. Because the deep‐pelagic domain has been a relatively stable habitat in terms of its size and temperature for millions of years, it would seem likely that the demographic histories of deep‐pelagic fishes would be characterized by a lack of expansions/contractions (Clark et al., 2009; Levitus et al., 2012; Robison, 2009). Instead, we uncovered a minimum of four cases of population expansion (identified by both frequency‐based statistics and gene tree‐based analysis) and possibly nine cases of population expansion (based on frequency‐based statistics alone).

We proposed and tested one potential driver of population size change in deep‐pelagic fishes, diel vertical migration. We hypothesized that the obligate use of the more volatile epipelagic domain by vertically migrating species may increase their likelihood of undergoing population fluctuations relative to species that do not vertically migrate. Hsieh et al. (2009) provide support for the hypothesis as they reported the larval distribution of fish species with vertically migrating adults changed more rapidly/frequently than non‐vertically migrating species (Hsieh et al., 2009). This could be attributed to short‐term changes in surface water conditions that impact vertically migrating species but are unfelt by those adults that remain at depth. If vertical migratory habits were the primary driver of population dynamics in deep‐pelagic fishes, the demographic histories of vertically migrating species would be characterized by population expansions/contractions, while non‐vertically migrating species should be less variable over time. Based on our chi‐squared test, we were unable to detect any difference in population dynamics between these two groups. Of the four species with the strongest evidence for population expansions, two are vertical migrators and two are non‐vertical migrators. We find vertical migration, to be an unlikely driver of population dynamics in deep‐pelagic fishes.

Another feature of deep‐pelagic fish biology might explain population dynamics in the fishes inhabiting this environment, a pelagic larval phase, where the larvae of most deep‐pelagic fishes reside in the upper 200 m (Bowlin, 2016; Johnson et al., 2009; Moser, 1996). Two lines of evidence support the hypothesis that the physiological tolerances of the larvae residing in the epipelagic domain drive population dynamics in deep‐pelagic fishes: long‐term monitoring of larval distribution and deep‐pelagic patterns of distribution.

Long‐term monitoring efforts in transition zones between tropical and subpolar regions have shown that physical conditions, such as sea surface temperature, are key predictors of larval community composition. Furthermore, physical changes in these environments alter the larval composition of the community (Ahlstrom, 1969; Netburn & Koslow, 2018; Sassa et al., 2004; Urias‐Leyva et al., 2018). Aceves‐Medina et al. (2004) found that the distribution of larvae was congruent with that of the adults. This suggests that as sea surface conditions alter larval distributions, the ranges of adults would change accordingly.

The second piece of evidence for larval control on demography comes from the distribution patterns of deep‐pelagic fishes. Most deep‐pelagic fishes can broadly be classified as warm‐water or cold‐water species, and many species have latitudinal biogeographic boundaries (see Table A3 for range description of study species) (Olson, 2001; Pearcy, 1991; Randall, 1981). Within oceanic basins, latitudinal differences in temperature decrease by depth (Vecchione et al., 2015). By 1000 m depth the temperature is a near uniform 5°C throughout most of the world's oceans (Helfman et al., 2009; Tyus, 2011). It is therefore noteworthy that even some non‐vertically migrating bathypelagic groups such as the whale fishes exhibit strong latitudinal biogeographic boundaries (Paxton, 1989). It seems unlikely that the distribution trends exhibited by deep‐pelagic fishes can be explained by physiological constraints on the adults of these species given the relative homogeneity of the environment. Rather, a given species range is constrained to regions with surface waters tolerable to their larvae. If correct periods of warm SST in high latitudes would increase available habitat to deep‐pelagic larval fishes and lead to population expansions.

Finally, trophic dynamics could potentially drive changes in population size in deep‐pelagic fishes. Vertically migrating fishes in the upper mesopelagic typically forage in surface waters where the food web is supported by recent in situ primary production (Gloeckler et al., 2018; Sutton & Hopkins, 1996). In contrast, some non‐migratory fishes that reside in the lower mesopelagic and the bathypelagic largely rely on a suspended, particle‐based food web composed of degraded particulate organic matter originating in the epipelagic (Crichton et al., 2023; Eduardo et al., 2023; Gloeckler et al., 2018; Hannides et al., 2020). Thus, the amount of carbon available to non‐migratory consumers in the lower mesopelagic and bathypelagic zones is directly linked to the amount of primary production in surface waters and the rate at which it can reach deeper depths.

Changes in ocean temperatures can greatly alter the amount of the particulate organic carbon (POC) that reaches this environment, in turn affecting the amount of food available to support this community (Crichton et al., 2023; John et al., 2013; Olivarez Lyle & Lyle, 2006). Crichton et al. (2023) suggest that bacteria may drive this phenomenon. As oceans warm the metabolic rates of bacteria increase, leading to greater consumption of sinking organic material, and less POC reaching deep waters (Crichton et al., 2023).

The availability of organic carbon at depth may be evident in the fossil record (Boscolo‐Galazzo et al., 2021). Deep‐pelagic formaninifera diversity and abundance increases as ocean temperatures decrease, a feature attributed to a greater volume of food reaching this habitat (Boscolo‐Galazzo et al., 2021; Crichton et al., 2023). This increase in food availability would be expected to positively benefit population sizes for the other members of the deep‐pelagic food web, including fish species. If food availability shapes deep‐pelagic fish demographics, periods of global warming would lead to population contractions while periods of global cooling would lead to population expansions. This feature would be more pronounced in deeper dwelling species.

## 5 CONCLUSIONS

Insights into the nature of deep‐pelagic fish population dynamics are currently lacking. Our results demonstrate that despite the long‐term stability of the global mesopelagic and bathypelagic domains, the population sizes of the fishes that reside within them are not static in nature. It seems likely that previous changes to the environment, potentially as a result of large‐scale changes in climate, have impacted the fish community residing in the deep‐pelagic. As we continue to investigate the particular environmental factors that influence demographic changes in these fishes, we will better be able to predict how populations of these fishes will behave in the face of future climate change.

## AUTHOR CONTRIBUTIONS

**Max D. Weber:** Conceptualization (lead); data curation (lead); investigation (equal); writing – original draft (lead). **Travis M. Richards:** Investigation (equal); writing – review and editing (equal). **Tracey T. Sutton:** Funding acquisition (lead); investigation (equal); writing – review and editing (equal). **Joshua E. Carter:** Investigation (equal); writing – review and editing (equal). **Ron I. Eytan:** Funding acquisition (supporting); investigation (equal); resources (lead); supervision (lead); writing – review and editing (equal).

## CONFLICT OF INTEREST STATEMENT

The authors declare that they have no conflicts of interest.

## Data Availability

DNA sequences: available on Genbank. The accession numbers and corresponding sample data are located in Table A1.

## Appendix

**TABLE A1:** List of GenBank accession numbers.

Species	Gene	Accession number	Specimen ID
*Bathophilus pawneei*	COI	OP131918	DPND_1336
*Bathophilus pawneei*	COI	OP131919	DPND_1900
*Bathophilus pawneei*	COI	OP131920	DPND_1914
*Bathophilus pawneei*	COI	OP131921	DPND_1985
*Bathophilus pawneei*	COI	OP131922	DPND_2001
*Bathophilus pawneei*	COI	OP131923	DPND_2002
*Bathophilus pawneei*	COI	OP131924	DPND_2267
*Bathophilus pawneei*	COI	OP131925	RIE_0008
*Bathophilus pawneei*	COI	OP131926	RIE_0313
*Bathophilus pawneei*	COI	OP131927	RIE_0516
*Chauliodus sloani*	COI	OP132420	DPND_1241
*Chauliodus sloani*	COI	OP132421	DPND_1556
*Chauliodus sloani*	COI	OP132422	DPND_1557
*Chauliodus sloani*	COI	OP132423	DPND_1605
*Chauliodus sloani*	COI	OP132424	DPND_1669
*Chauliodus sloani*	COI	OP132425	DPND_1695
*Chauliodus sloani*	COI	OP132426	DPND_1877
*Chauliodus sloani*	COI	OP132427	DPND_1895
*Chauliodus sloani*	COI	OP132428	DPND_1896
*Chauliodus sloani*	COI	OP132429	DPND_1937
*Chauliodus sloani*	COI	OP132430	DPND_1997
*Chauliodus sloani*	COI	OP132431	DPND_2027
*Chauliodus sloani*	COI	OP132432	DPND_2037
*Chauliodus sloani*	COI	OP132433	DPND_2097
*Chauliodus sloani*	COI	OP132434	DPND_2181
*Chauliodus sloani*	COI	OP132435	DPND_2208
*Chauliodus sloani*	COI	OP132436	DPND_2260
*Chauliodus sloani*	COI	OP132437	DPND_2387
*Chauliodus sloani*	COI	OP132438	DPND_2418
*Chauliodus sloani*	COI	OP132439	DPND_2490
*Chauliodus sloani*	COI	OP132440	DPND_2528
*Chauliodus sloani*	COI	OP132441	DPND_2699
*Chauliodus sloani*	COI	OP132442	DPND_2731
*Chauliodus sloani*	COI	OP132443	DPND_2756
*Chauliodus sloani*	COI	OP132444	DPND_2757
*Chauliodus sloani*	COI	OP132445	DPND_2813
*Chauliodus sloani*	COI	OP132446	DPND_2958
*Chauliodus sloani*	COI	OP132447	DPND_3621
*Chauliodus sloani*	COI	OP132448	DPND_3682
*Chauliodus sloani*	COI	OP132449	DPND_3774
*Chauliodus sloani*	COI	OP132450	DPND_3789
*Chauliodus sloani*	COI	OP132451	DPND_3790
*Chauliodus sloani*	COI	OP132452	DPND_3937
*Chauliodus sloani*	COI	OP132453	DPND_3938
*Chauliodus sloani*	COI	OP132454	DPND_4109
*Chauliodus sloani*	COI	OP132455	DPND_4119
*Chauliodus sloani*	COI	OP132456	DPND_4120
*Chauliodus sloani*	COI	OP132457	DPND_4121
*Chauliodus sloani*	COI	OP132458	DPND_4138
*Chauliodus sloani*	COI	OP132459	DPND_4139
*Chauliodus sloani*	COI	OP132460	DPND_4212
*Chauliodus sloani*	COI	OP132461	DPND_4278
*Chauliodus sloani*	COI	OP132462	DPND_4465
*Chauliodus sloani*	COI	OP132463	DPND_4528
*Chauliodus sloani*	COI	OP132464	DPND_4529
*Chauliodus sloani*	COI	OP132465	DPND_4546
*Chauliodus sloani*	COI	OP132466	DPND_4551
*Chauliodus sloani*	COI	OP132467	DPND_4649
*Chauliodus sloani*	COI	OP132468	DPND_4693
*Chauliodus sloani*	COI	OP132469	DPND_4694
*Chauliodus sloani*	COI	OP132470	DPND_4695
*Chauliodus sloani*	COI	OP132471	DPND_4736
*Chauliodus sloani*	COI	OP132472	DPND_4920
*Chauliodus sloani*	COI	OP132473	DPND_4946
*Chauliodus sloani*	COI	OP132474	DPND_5014
*Chauliodus sloani*	COI	OP132475	DPND_5015
*Chauliodus sloani*	COI	OP132476	DPND_5050
*Chauliodus sloani*	COI	OP132477	DPND_5126
*Chauliodus sloani*	COI	OP132478	DPND_5302
*Chauliodus sloani*	COI	OP132479	DPND_5334
*Chauliodus sloani*	COI	OP132480	DPND_5335
*Chauliodus sloani*	COI	OP132481	DPND_5398
*Chauliodus sloani*	COI	OP132482	DPND_5426
*Chauliodus sloani*	COI	OP132483	DPND_5427
*Chauliodus sloani*	COI	OP132484	DPND_5467
*Chauliodus sloani*	COI	OP132485	DPND_5468
*Chauliodus sloani*	COI	OP132486	DPND_5500
*Chauliodus sloani*	COI	OP132487	DPND_5573
*Chauliodus sloani*	COI	OP132488	DPND_5574
*Chauliodus sloani*	COI	OP132489	DPND_5593
*Chauliodus sloani*	COI	OP132490	DPND_5610
*Chauliodus sloani*	COI	OP132491	DPND_5634
*Chauliodus sloani*	COI	OP132492	DPND_5665
*Chauliodus sloani*	COI	OP132493	DPND_5675
*Chauliodus sloani*	COI	OP132494	DPND_5680
*Chauliodus sloani*	COI	OP132495	DPND_5682
*Chauliodus sloani*	COI	OP132496	DPND_5723
*Chauliodus sloani*	COI	OP132497	DPND_5741
*Chauliodus sloani*	COI	OP132498	RIE_18
*Chauliodus sloani*	COI	OP132499	RIE_236
*Chauliodus sloani*	COI	OP132500	RIE_510
*Chauliodus sloani*	COI	OP132501	RIE_576
*Chauliodus sloani*	COI	OP132502	RIE_642
*Chauliodus sloani*	COI	OP132503	RIE_690
*Chauliodus sloani*	COI	OP132504	RIE_698
*Chauliodus sloani*	COI	OP132505	RIE_912
*Chauliodus sloani*	COI	OP132506	RIE_913
*Chauliodus sloani*	COI	OP132507	RIE_914
*Chauliodus sloani*	COI	OP132508	RIE_948
*Chauliodus sloani*	COI	OP132509	RIE_0994
*Chauliodus sloani*	COI	OP132510	DPND_5114
*Chauliodus sloani*	COI	OP132511	DPND_4579
*Chauliodus sloani*	COI	OP132512	DPND_2788
*Chauliodus sloani*	COI	OP132513	DPND_3505
*Chauliodus sloani*	COI	OP132514	DPND_4717
*Chauliodus sloani*	COI	OP132515	DPND_4008
*Chauliodus sloani*	COI	OP132516	DPND_3987
*Cyclothone alba*	COI	OP131971	RIE_0349
*Cyclothone alba*	COI	OP131972	RIE_0350
*Cyclothone alba*	COI	OP131973	RIE_0351
*Cyclothone alba*	COI	OP131974	RIE_0031
*Cyclothone alba*	COI	OP131975	RIE_0252
*Cyclothone alba*	COI	OP131976	RIE_0253
*Cyclothone alba*	COI	OP131977	RIE_0254
*Cyclothone alba*	COI	OP131978	RIE_0255
*Cyclothone alba*	COI	OP131979	RIE_0344
*Cyclothone alba*	COI	OP131980	RIE_0345
*Cyclothone alba*	COI	OP131981	RIE_0347
*Cyclothone alba*	COI	OP131982	RIE_0348
*Cyclothone pseudopallida*	COI	OP131984	RIE_0359
*Cyclothone pseudopallida*	COI	OP131985	RIE_0357
*Cyclothone pseudopallida*	COI	OP131986	RIE_0077
*Cyclothone pseudopallida*	COI	OP131987	RIE_0354
*Cyclothone pseudopallida*	COI	OP131988	RIE_0239
*Cyclothone pseudopallida*	COI	OP131989	RIE_0360
*Cyclothone pseudopallida*	COI	OP131990	RIE_0477
*Cyclothone pseudopallida*	COI	OP131991	RIE_0493
*Cyclothone pseudopallida*	COI	OP131992	RIE_0544
*Cyclothone pseudopallida*	COI	OP131993	DPND_4547
*Cyclothone pseudopallida*	COI	OP131994	DPND_4483
*Cyclothone pseudopallida*	COI	OP131995	RIE_0238
*Cyclothone pseudopallida*	COI	OP131996	RIE_0358
*Cyclothone pseudopallida*	COI	OP131997	RIE_0492
*Diplospinus multistriatus*	COI	OP132142	DPND_2718
*Diplospinus multistriatus*	COI	OP132143	RIE_0886
*Diplospinus multistriatus*	COI	OP132144	RIE_0887
*Diplospinus multistriatus*	COI	OP132145	RIE_0171
*Diplospinus multistriatus*	COI	OP132146	RIE_0240
*Diplospinus multistriatus*	COI	OP132147	RIE_0413
*Diplospinus multistriatus*	COI	OP132148	RIE_0495
*Diplospinus multistriatus*	COI	OP132149	RIE_0713
*Diplospinus multistriatus*	COI	OP132150	RIE_0882
*Diplospinus multistriatus*	COI	OP132151	RIE_0883
*Diplospinus multistriatus*	COI	OP132152	RIE_0884
*Diplospinus multistriatus*	COI	OP132153	RIE_0885
*Ditropichthys storeri*	COI	OP131959	DPND_2989
*Ditropichthys storeri*	COI	OP131960	DDPND_3302
*Ditropichthys storeri*	COI	OP131961	DPND_3911
*Ditropichthys storeri*	COI	OP131962	DPND_4130
*Ditropichthys storeri*	COI	OP131963	DPND_4210
*Ditropichthys storeri*	COI	OP131964	DPND_1466
*Ditropichthys storeri*	COI	OP131965	DPND_2251
*Ditropichthys storeri*	COI	OP131966	RIE_0438
*Ditropichthys storeri*	COI	OP131967	RIE_0522
*Ditropichthys storeri*	COI	OP131968	RIE_0243
*Ditropichthys storeri*	COI	OP131969	DPND_4431
*Photostomias guernei*	COI	OP132211	RIE_0506
*Photostomias guernei*	COI	OP132212	RIE_0514
*Photostomias guernei*	COI	OP132213	RIE_0551
*Photostomias guernei*	COI	OP132214	RIE_0081
*Photostomias guernei*	COI	OP132215	RIE_0107
*Photostomias guernei*	COI	OP132216	RIE_0176
*Photostomias guernei*	COI	OP132217	RIE_0337
*Photostomias guernei*	COI	OP132218	RIE_0400
*Photostomias guernei*	COI	OP132219	RIE_0401
*Photostomias guernei*	COI	OP132220	RIE_0459
*Photostomias guernei*	COI	OP132221	RIE_0460
*Photostomias guernei*	COI	OP132222	RIE_0461
*Scopelogadus mizolepis*	COI	OP132154	DPND_2511
*Scopelogadus mizolepis*	COI	OP132155	DPND_2512
*Scopelogadus mizolepis*	COI	OP132156	DPND_4271
*Scopelogadus mizolepis*	COI	OP132157	DPND_1298
*Scopelogadus mizolepis*	COI	OP132158	DPND_1299
*Scopelogadus mizolepis*	COI	OP132159	DPND_1379
*Scopelogadus mizolepis*	COI	OP132160	DPND_1380
*Scopelogadus mizolepis*	COI	OP132161	DPND_2220
*Scopelogadus mizolepis*	COI	OP132162	RIE_0041
*Scopelogadus mizolepis*	COI	OP132163	RIE_0518
*Scopelogadus mizolepis*	COI	OP132164	RIE_0954
*Sigmops elongatus*	COI	OP132179	RIE_0278
*Sigmops elongatus*	COI	OP132180	RIE_0279
*Sigmops elongatus*	COI	OP132181	RIE_0280
*Sigmops elongatus*	COI	OP132182	RIE_0002
*Sigmops elongatus*	COI	OP132183	RIE_0017
*Sigmops elongatus*	COI	OP132184	RIE_0097
*Sigmops elongatus*	COI	OP132185	RIE_0182
*Sigmops elongatus*	COI	OP132186	RIE_0204
*Sigmops elongatus*	COI	OP132187	RIE_0205
*Sigmops elongatus*	COI	OP132188	RIE_0206
*Sigmops elongatus*	COI	OP132189	RIE_0244
*Sigmops elongatus*	COI	OP132190	RIE_0277
*Sternoptyx pseudobscura*	COI	OP132242	RIE_0415
*Sternoptyx pseudobscura*	COI	OP132243	RIE_0417
*Sternoptyx pseudobscura*	COI	OP132244	RIE_0078
*Sternoptyx pseudobscura*	COI	OP132245	RIE_0195
*Sternoptyx pseudobscura*	COI	OP132246	RIE_0196
*Sternoptyx pseudobscura*	COI	OP132247	RIE_0197
*Sternoptyx pseudobscura*	COI	OP132248	RIE_0200
*Sternoptyx pseudobscura*	COI	OP132249	RIE_0201
*Sternoptyx pseudobscura*	COI	OP132250	RIE_0223
*Sternoptyx pseudobscura*	COI	OP132251	RIE_0224
*Sternoptyx pseudobscura*	COI	OP132252	RIE_0226
*Sternoptyx pseudobscura*	COI	OP132253	DPND_3772
*Sternoptyx pseudobscura*	COI	OP132254	DPND_4225
*Stomias affinis*	COI	OP132165	RIE_0237
*Stomias affinis*	COI	OP132166	RIE_0517
*Stomias affinis*	COI	OP132167	DPND_3645
*Stomias affinis*	COI	OP132168	DPND_1314
*Stomias affinis*	COI	OP132169	DPND_1543
*Stomias affinis*	COI	OP132170	DPND_1639
*Stomias affinis*	COI	OP132171	RIE_0917
*Stomias affinis*	COI	OP132172	RIE_0466
*Stomias affinis*	COI	OP132173	RIE_0577
*Stomias affinis*	COI	OP132174	DPND_1201
*Stomias affinis*	COI	OP132175	DPND_1315
*Bathophilus pawneei*	PLAG1	OP149759	DPND_1336
*Bathophilus pawneei*	PLAG1	OP149760	DPND_1336
*Bathophilus pawneei*	PLAG1	OP149761	DPND_1900
*Bathophilus pawneei*	PLAG1	OP149762	DPND_1900
*Bathophilus pawneei*	PLAG1	OP149763	DPND_1914
*Bathophilus pawneei*	PLAG1	OP149764	DPND_1914
*Bathophilus pawneei*	PLAG1	OP149765	DPND_1985
*Bathophilus pawneei*	PLAG1	OP149766	DPND_1985
*Bathophilus pawneei*	PLAG1	OP149767	DPND_2001
*Bathophilus pawneei*	PLAG1	OP149768	DPND_2001
*Bathophilus pawneei*	PLAG1	OP149769	DPND_2002
*Bathophilus pawneei*	PLAG1	OP149770	DPND_2002
*Bathophilus pawneei*	PLAG1	OP149771	DPND_2267
*Bathophilus pawneei*	PLAG1	OP149772	DPND_2267
*Bathophilus pawneei*	PLAG1	OP149773	RIE_313
*Bathophilus pawneei*	PLAG1	OP149774	RIE_313
*Bathophilus pawneei*	PLAG1	OP149775	RIE_516
*Bathophilus pawneei*	PLAG1	OP149776	RIE_516
*Bathophilus pawneei*	PLAG1	OP149777	RIE_8
*Bathophilus pawneei*	PLAG1	OP149778	RIE_8
*Cyclothone alba*	PLAG1	OP149779	RIE_252
*Cyclothone alba*	PLAG1	OP149780	RIE_252
*Cyclothone alba*	PLAG1	OP149781	RIE_253
*Cyclothone alba*	PLAG1	OP149782	RIE_253
*Cyclothone alba*	PLAG1	OP149783	RIE_254
*Cyclothone alba*	PLAG1	OP149784	RIE_254
*Cyclothone alba*	PLAG1	OP149785	RIE_255
*Cyclothone alba*	PLAG1	OP149786	RIE_255
*Cyclothone alba*	PLAG1	OP149787	RIE_31
*Cyclothone alba*	PLAG1	OP149788	RIE_31
*Cyclothone alba*	PLAG1	OP149789	RIE_344
*Cyclothone alba*	PLAG1	OP149790	RIE_344
*Cyclothone alba*	PLAG1	OP149791	RIE_345
*Cyclothone alba*	PLAG1	OP149792	RIE_345
*Cyclothone alba*	PLAG1	OP149793	RIE_347
*Cyclothone alba*	PLAG1	OP149794	RIE_347
*Cyclothone alba*	PLAG1	OP149795	RIE_348
*Cyclothone alba*	PLAG1	OP149796	RIE_348
*Cyclothone alba*	PLAG1	OP149797	RIE_349
*Cyclothone alba*	PLAG1	OP149798	RIE_349
*Cyclothone alba*	PLAG1	OP149799	RIE_350
*Cyclothone alba*	PLAG1	OP149800	RIE_350
*Cyclothone alba*	PLAG1	OP149801	RIE_351
*Cyclothone alba*	PLAG1	OP149802	RIE_351
*Cyclothone pseudopallida*	PLAG1	OP149803	RIE_238
*Cyclothone pseudopallida*	PLAG1	OP149804	RIE_238
*Cyclothone pseudopallida*	PLAG1	OP149805	RIE_239
*Cyclothone pseudopallida*	PLAG1	OP149806	RIE_239
*Cyclothone pseudopallida*	PLAG1	OP149807	RIE_357
*Cyclothone pseudopallida*	PLAG1	OP149808	RIE_357
*Cyclothone pseudopallida*	PLAG1	OP149809	RIE_358
*Cyclothone pseudopallida*	PLAG1	OP149810	RIE_358
*Cyclothone pseudopallida*	PLAG1	OP149811	RIE_359
*Cyclothone pseudopallida*	PLAG1	OP149812	RIE_359
*Cyclothone pseudopallida*	PLAG1	OP149813	RIE_360
*Cyclothone pseudopallida*	PLAG1	OP149814	RIE_360
*Cyclothone pseudopallida*	PLAG1	OP149815	RIE_477
*Cyclothone pseudopallida*	PLAG1	OP149816	RIE_477
*Cyclothone pseudopallida*	PLAG1	OP149817	RIE_492
*Cyclothone pseudopallida*	PLAG1	OP149818	RIE_492
*Cyclothone pseudopallida*	PLAG1	OP149819	RIE_493
*Cyclothone pseudopallida*	PLAG1	OP149820	RIE_493
*Cyclothone pseudopallida*	PLAG1	OP149821	RIE_544
*Cyclothone pseudopallida*	PLAG1	OP149822	RIE_544
*Diplospinus multistriatus*	PLAG1	OP149823	DPND_2718
*Diplospinus multistriatus*	PLAG1	OP149824	DPND_2718
*Diplospinus multistriatus*	PLAG1	OP149825	RIE_171
*Diplospinus multistriatus*	PLAG1	OP149826	RIE_171
*Diplospinus multistriatus*	PLAG1	OP149827	RIE_240
*Diplospinus multistriatus*	PLAG1	OP149828	RIE_240
*Diplospinus multistriatus*	PLAG1	OP149829	RIE_413
*Diplospinus multistriatus*	PLAG1	OP149830	RIE_413
*Diplospinus multistriatus*	PLAG1	OP149831	RIE_495
*Diplospinus multistriatus*	PLAG1	OP149832	RIE_495
*Diplospinus multistriatus*	PLAG1	OP149833	RIE_713
*Diplospinus multistriatus*	PLAG1	OP149834	RIE_713
*Diplospinus multistriatus*	PLAG1	OP149835	RIE_882
*Diplospinus multistriatus*	PLAG1	OP149836	RIE_882
*Diplospinus multistriatus*	PLAG1	OP149837	RIE_883
*Diplospinus multistriatus*	PLAG1	OP149838	RIE_883
*Diplospinus multistriatus*	PLAG1	OP149839	RIE_884
*Diplospinus multistriatus*	PLAG1	OP149840	RIE_884
*Diplospinus multistriatus*	PLAG1	OP149841	RIE_885
*Diplospinus multistriatus*	PLAG1	OP149842	RIE_885
*Diplospinus multistriatus*	PLAG1	OP149843	RIE_886
*Diplospinus multistriatus*	PLAG1	OP149844	RIE_886
*Diplospinus multistriatus*	PLAG1	OP149845	RIE_887
*Diplospinus multistriatus*	PLAG1	OP149846	RIE_887
*Ditropichthys storeri*	PLAG1	OP149847	DPND_1466
*Ditropichthys storeri*	PLAG1	OP149848	DPND_1466
*Ditropichthys storeri*	PLAG1	OP149849	DPND_2251
*Ditropichthys storeri*	PLAG1	OP149850	DPND_2251
*Ditropichthys storeri*	PLAG1	OP149851	DPND_2989
*Ditropichthys storeri*	PLAG1	OP149852	DPND_2989
*Ditropichthys storeri*	PLAG1	OP149853	DPND_3302
*Ditropichthys storeri*	PLAG1	OP149854	DPND_3302
*Ditropichthys storeri*	PLAG1	OP149855	DPND_3911
*Ditropichthys storeri*	PLAG1	OP149856	DPND_3911
*Ditropichthys storeri*	PLAG1	OP149857	DPND_4130
*Ditropichthys storeri*	PLAG1	OP149858	DPND_4130
*Ditropichthys storeri*	PLAG1	OP149859	DPND_4210
*Ditropichthys storeri*	PLAG1	OP149860	DPND_4210
*Ditropichthys storeri*	PLAG1	OP149861	RIE_243
*Ditropichthys storeri*	PLAG1	OP149862	RIE_243
*Ditropichthys storeri*	PLAG1	OP149863	RIE_438
*Ditropichthys storeri*	PLAG1	OP149864	RIE_438
*Ditropichthys storeri*	PLAG1	OP149865	RIE_522
*Ditropichthys storeri*	PLAG1	OP149866	RIE_522
*Photostomias guernei*	PLAG1	OP149867	RIE_107
*Photostomias guernei*	PLAG1	OP149868	RIE_107
*Photostomias guernei*	PLAG1	OP149869	RIE_176
*Photostomias guernei*	PLAG1	OP149870	RIE_176
*Photostomias guernei*	PLAG1	OP149871	RIE_337
*Photostomias guernei*	PLAG1	OP149872	RIE_337
*Photostomias guernei*	PLAG1	OP149873	RIE_400
*Photostomias guernei*	PLAG1	OP149874	RIE_400
*Photostomias guernei*	PLAG1	OP149875	RIE_401
*Photostomias guernei*	PLAG1	OP149876	RIE_401
*Photostomias guernei*	PLAG1	OP149877	RIE_459
*Photostomias guernei*	PLAG1	OP149878	RIE_459
*Photostomias guernei*	PLAG1	OP149879	RIE_461
*Photostomias guernei*	PLAG1	OP149880	RIE_461
*Photostomias guernei*	PLAG1	OP149881	RIE_506
*Photostomias guernei*	PLAG1	OP149882	RIE_506
*Photostomias guernei*	PLAG1	OP149883	RIE_514
*Photostomias guernei*	PLAG1	OP149884	RIE_514
*Photostomias guernei*	PLAG1	OP149885	RIE_551
*Photostomias guernei*	PLAG1	OP149886	RIE_551
*Photostomias guernei*	PLAG1	OP149887	RIE_552
*Photostomias guernei*	PLAG1	OP149888	RIE_552
*Photostomias guernei*	PLAG1	OP149889	RIE_81
*Photostomias guernei*	PLAG1	OP149890	RIE_81
*Scopelogadus mizolepis*	PLAG1	OP149891	DPND_1298
*Scopelogadus mizolepis*	PLAG1	OP149892	DPND_1298
*Scopelogadus mizolepis*	PLAG1	OP149893	DPND_1299
*Scopelogadus mizolepis*	PLAG1	OP149894	DPND_1299
*Scopelogadus mizolepis*	PLAG1	OP149895	DPND_1379
*Scopelogadus mizolepis*	PLAG1	OP149896	DPND_1379
*Scopelogadus mizolepis*	PLAG1	OP149897	DPND_1380
*Scopelogadus mizolepis*	PLAG1	OP149898	DPND_1380
*Scopelogadus mizolepis*	PLAG1	OP149899	DPND_2220
*Scopelogadus mizolepis*	PLAG1	OP149900	DPND_2220
*Scopelogadus mizolepis*	PLAG1	OP149901	DPND_2511
*Scopelogadus mizolepis*	PLAG1	OP149902	DPND_2511
*Scopelogadus mizolepis*	PLAG1	OP149903	DPND_2512
*Scopelogadus mizolepis*	PLAG1	OP149904	DPND_2512
*Scopelogadus mizolepis*	PLAG1	OP149905	DPND_4271
*Scopelogadus mizolepis*	PLAG1	OP149906	DPND_4271
*Scopelogadus mizolepis*	PLAG1	OP149907	RIE_41
*Scopelogadus mizolepis*	PLAG1	OP149908	RIE_41
*Scopelogadus mizolepis*	PLAG1	OP149909	RIE_518
*Scopelogadus mizolepis*	PLAG1	OP149910	RIE_518
*Scopelogadus mizolepis*	PLAG1	OP149911	RIE_954
*Scopelogadus mizolepis*	PLAG1	OP149912	RIE_954
*Sigmops elongatus*	PLAG1	OP149913	RIE_17
*Sigmops elongatus*	PLAG1	OP149914	RIE_17
*Sigmops elongatus*	PLAG1	OP149915	RIE_182
*Sigmops elongatus*	PLAG1	OP149916	RIE_182
*Sigmops elongatus*	PLAG1	OP149917	RIE_204
*Sigmops elongatus*	PLAG1	OP149918	RIE_204
*Sigmops elongatus*	PLAG1	OP149919	RIE_205
*Sigmops elongatus*	PLAG1	OP149920	RIE_205
*Sigmops elongatus*	PLAG1	OP149921	RIE_206
*Sigmops elongatus*	PLAG1	OP149922	RIE_206
*Sigmops elongatus*	PLAG1	OP149923	RIE_244
*Sigmops elongatus*	PLAG1	OP149924	RIE_244
*Sigmops elongatus*	PLAG1	OP149925	RIE_277
*Sigmops elongatus*	PLAG1	OP149926	RIE_277
*Sigmops elongatus*	PLAG1	OP149927	RIE_278
*Sigmops elongatus*	PLAG1	OP149928	RIE_278
*Sigmops elongatus*	PLAG1	OP149929	RIE_279
*Sigmops elongatus*	PLAG1	OP149930	RIE_279
*Sigmops elongatus*	PLAG1	OP149931	RIE_280
*Sigmops elongatus*	PLAG1	OP149932	RIE_280
*Sigmops elongatus*	PLAG1	OP149933	RIE_2
*Sigmops elongatus*	PLAG1	OP149934	RIE_2
*Sigmops elongatus*	PLAG1	OP149935	RIE_97
*Sigmops elongatus*	PLAG1	OP149936	RIE_97
*Sternoptyx pseudobscura*	PLAG1	OP149937	DPND_3772
*Sternoptyx pseudobscura*	PLAG1	OP149938	DPND_3772
*Sternoptyx pseudobscura*	PLAG1	OP149939	RIE_195
*Sternoptyx pseudobscura*	PLAG1	OP149940	RIE_195
*Sternoptyx pseudobscura*	PLAG1	OP149941	RIE_196
*Sternoptyx pseudobscura*	PLAG1	OP149942	RIE_196
*Sternoptyx pseudobscura*	PLAG1	OP149943	RIE_197
*Sternoptyx pseudobscura*	PLAG1	OP149944	RIE_197
*Sternoptyx pseudobscura*	PLAG1	OP149945	RIE_200
*Sternoptyx pseudobscura*	PLAG1	OP149946	RIE_200
*Sternoptyx pseudobscura*	PLAG1	OP149947	RIE_201
*Sternoptyx pseudobscura*	PLAG1	OP149948	RIE_201
*Sternoptyx pseudobscura*	PLAG1	OP149949	RIE_223
*Sternoptyx pseudobscura*	PLAG1	OP149950	RIE_223
*Sternoptyx pseudobscura*	PLAG1	OP149951	RIE_224
*Sternoptyx pseudobscura*	PLAG1	OP149952	RIE_224
*Sternoptyx pseudobscura*	PLAG1	OP149953	RIE_225
*Sternoptyx pseudobscura*	PLAG1	OP149954	RIE_225
*Sternoptyx pseudobscura*	PLAG1	OP149955	RIE_226
*Sternoptyx pseudobscura*	PLAG1	OP149956	RIE_226
*Sternoptyx pseudobscura*	PLAG1	OP149957	RIE_415
*Sternoptyx pseudobscura*	PLAG1	OP149958	RIE_415
*Sternoptyx pseudobscura*	PLAG1	OP149959	RIE_417
*Sternoptyx pseudobscura*	PLAG1	OP149960	RIE_417
*Sternoptyx pseudobscura*	PLAG1	OP149961	RIE_537
*Sternoptyx pseudobscura*	PLAG1	OP149962	RIE_537
*Sternoptyx pseudobscura*	PLAG1	OP149963	RIE_538
*Sternoptyx pseudobscura*	PLAG1	OP149964	RIE_538
*Sternoptyx pseudobscura*	PLAG1	OP149965	RIE_539
*Sternoptyx pseudobscura*	PLAG1	OP149966	RIE_539
*Sternoptyx pseudobscura*	PLAG1	OP149967	RIE_78
*Sternoptyx pseudobscura*	PLAG1	OP149968	RIE_78
*Sternoptyx pseudobscura*	PLAG1	OP149969	RIE_915
*Sternoptyx pseudobscura*	PLAG1	OP149970	RIE_915
*Chauliodus sloani*	ENC1	OP149971	DPND_1556
*Chauliodus sloani*	ENC1	OP149972	DPND_1556
*Chauliodus sloani*	ENC1	OP149973	DPND_1669
*Chauliodus sloani*	ENC1	OP149974	DPND_1669
*Chauliodus sloani*	ENC1	OP149975	DPND_1695
*Chauliodus sloani*	ENC1	OP149976	DPND_1695
*Chauliodus sloani*	ENC1	OP149977	DPND_1877
*Chauliodus sloani*	ENC1	OP149978	DPND_1877
*Chauliodus sloani*	ENC1	OP149979	DPND_1895
*Chauliodus sloani*	ENC1	OP149980	DPND_1895
*Chauliodus sloani*	ENC1	OP149981	DPND_1896
*Chauliodus sloani*	ENC1	OP149982	DPND_1896
*Chauliodus sloani*	ENC1	OP149983	DPND_1937
*Chauliodus sloani*	ENC1	OP149984	DPND_1937
*Chauliodus sloani*	ENC1	OP149985	DPND_2027
*Chauliodus sloani*	ENC1	OP149986	DPND_2027
*Chauliodus sloani*	ENC1	OP149987	DPND_2037
*Chauliodus sloani*	ENC1	OP149988	DPND_2037
*Chauliodus sloani*	ENC1	OP149989	DPND_2097
*Chauliodus sloani*	ENC1	OP149990	DPND_2097
*Chauliodus sloani*	ENC1	OP149991	DPND_2208
*Chauliodus sloani*	ENC1	OP149992	DPND_2208
*Chauliodus sloani*	ENC1	OP149993	DPND_2260
*Chauliodus sloani*	ENC1	OP149994	DPND_2260
*Chauliodus sloani*	ENC1	OP149995	DPND_2387
*Chauliodus sloani*	ENC1	OP149996	DPND_2387
*Chauliodus sloani*	ENC1	OP149997	DPND_2418
*Chauliodus sloani*	ENC1	OP149998	DPND_2418
*Chauliodus sloani*	ENC1	OP149999	DPND_2490
*Chauliodus sloani*	ENC1	OP150000	DPND_2490
*Chauliodus sloani*	ENC1	OP150001	DPND_2528
*Chauliodus sloani*	ENC1	OP150002	DPND_2528
*Chauliodus sloani*	ENC1	OP150003	DPND_2669
*Chauliodus sloani*	ENC1	OP150004	DPND_2669
*Chauliodus sloani*	ENC1	OP150005	DPND_2756
*Chauliodus sloani*	ENC1	OP150006	DPND_2756
*Chauliodus sloani*	ENC1	OP150007	DPND_2757
*Chauliodus sloani*	ENC1	OP150008	DPND_2757
*Diplospinus multistriatus*	ENC1	OP150009	DPND_2718
*Diplospinus multistriatus*	ENC1	OP150010	DPND_2718
*Diplospinus multistriatus*	ENC1	OP150011	RIE_171
*Diplospinus multistriatus*	ENC1	OP150012	RIE_171
*Diplospinus multistriatus*	ENC1	OP150013	RIE_240
*Diplospinus multistriatus*	ENC1	OP150014	RIE_240
*Diplospinus multistriatus*	ENC1	OP150015	RIE_413
*Diplospinus multistriatus*	ENC1	OP150016	RIE_413
*Diplospinus multistriatus*	ENC1	OP150017	RIE_495
*Diplospinus multistriatus*	ENC1	OP150018	RIE_495
*Diplospinus multistriatus*	ENC1	OP150019	RIE_713
*Diplospinus multistriatus*	ENC1	OP150020	RIE_713
*Diplospinus multistriatus*	ENC1	OP150021	RIE_882
*Diplospinus multistriatus*	ENC1	OP150022	RIE_882
*Diplospinus multistriatus*	ENC1	OP150023	RIE_883
*Diplospinus multistriatus*	ENC1	OP150024	RIE_883
*Diplospinus multistriatus*	ENC1	OP150025	RIE_884
*Diplospinus multistriatus*	ENC1	OP150026	RIE_884
*Diplospinus multistriatus*	ENC1	OP150027	RIE_885
*Diplospinus multistriatus*	ENC1	OP150028	RIE_885
*Diplospinus multistriatus*	ENC1	OP150029	RIE_886
*Diplospinus multistriatus*	ENC1	OP150030	RIE_886
*Diplospinus multistriatus*	ENC1	OP150031	RIE_887
*Diplospinus multistriatus*	ENC1	OP150032	RIE_887
*Sternoptyx pseudobscura*	ENC1	OP150033	DPND_3772
*Sternoptyx pseudobscura*	ENC1	OP150034	DPND_3772
*Sternoptyx pseudobscura*	ENC1	OP150035	RIE_195
*Sternoptyx pseudobscura*	ENC1	OP150036	RIE_195
*Sternoptyx pseudobscura*	ENC1	OP150037	RIE_196
*Sternoptyx pseudobscura*	ENC1	OP150038	RIE_196
*Sternoptyx pseudobscura*	ENC1	OP150039	RIE_197
*Sternoptyx pseudobscura*	ENC1	OP150040	RIE_197
*Sternoptyx pseudobscura*	ENC1	OP150041	RIE_200
*Sternoptyx pseudobscura*	ENC1	OP150042	RIE_200
*Sternoptyx pseudobscura*	ENC1	OP150043	RIE_201
*Sternoptyx pseudobscura*	ENC1	OP150044	RIE_201
*Sternoptyx pseudobscura*	ENC1	OP150045	RIE_224
*Sternoptyx pseudobscura*	ENC1	OP150046	RIE_224
*Sternoptyx pseudobscura*	ENC1	OP150047	RIE_225
*Sternoptyx pseudobscura*	ENC1	OP150048	RIE_225
*Sternoptyx pseudobscura*	ENC1	OP150049	RIE_226
*Sternoptyx pseudobscura*	ENC1	OP150050	RIE_226
*Sternoptyx pseudobscura*	ENC1	OP150051	RIE_415
*Sternoptyx pseudobscura*	ENC1	OP150052	RIE_415
*Sternoptyx pseudobscura*	ENC1	OP150053	RIE_417
*Sternoptyx pseudobscura*	ENC1	OP150054	RIE_417
*Sternoptyx pseudobscura*	ENC1	OP150055	RIE_537
*Sternoptyx pseudobscura*	ENC1	OP150056	RIE_537
*Sternoptyx pseudobscura*	ENC1	OP150057	RIE_538
*Sternoptyx pseudobscura*	ENC1	OP150058	RIE_538
*Sternoptyx pseudobscura*	ENC1	OP150059	RIE_539
*Sternoptyx pseudobscura*	ENC1	OP150060	RIE_539
*Sternoptyx pseudobscura*	ENC1	OP150061	RIE_78
*Sternoptyx pseudobscura*	ENC1	OP150062	RIE_78
*Sternoptyx pseudobscura*	ENC1	OP150063	RIE_915
*Sternoptyx pseudobscura*	ENC1	OP150064	RIE_915
*Sternoptyx pseudobscura*	MYH6	OP150065	DPND_4225
*Sternoptyx pseudobscura*	MYH6	OP150066	DPND_4225
*Sternoptyx pseudobscura*	MYH6	OP150067	RIE_195
*Sternoptyx pseudobscura*	MYH6	OP150068	RIE_195
*Sternoptyx pseudobscura*	MYH6	OP150069	RIE_196
*Sternoptyx pseudobscura*	MYH6	OP150070	RIE_196
*Sternoptyx pseudobscura*	MYH6	OP150071	RIE_197
*Sternoptyx pseudobscura*	MYH6	OP150072	RIE_197
*Sternoptyx pseudobscura*	MYH6	OP150073	RIE_200
*Sternoptyx pseudobscura*	MYH6	OP150074	RIE_200
*Sternoptyx pseudobscura*	MYH6	OP150075	RIE_201
*Sternoptyx pseudobscura*	MYH6	OP150076	RIE_201
*Sternoptyx pseudobscura*	MYH6	OP150077	RIE_223
*Sternoptyx pseudobscura*	MYH6	OP150078	RIE_223
*Sternoptyx pseudobscura*	MYH6	OP150079	RIE_224
*Sternoptyx pseudobscura*	MYH6	OP150080	RIE_224
*Sternoptyx pseudobscura*	MYH6	OP150081	RIE_226
*Sternoptyx pseudobscura*	MYH6	OP150082	RIE_226
*Sternoptyx pseudobscura*	MYH6	OP150083	RIE_415
*Sternoptyx pseudobscura*	MYH6	OP150084	RIE_415
*Sternoptyx pseudobscura*	MYH6	OP150085	RIE_417
*Sternoptyx pseudobscura*	MYH6	OP150086	RIE_417
*Sternoptyx pseudobscura*	MYH6	OP150087	RIE_537
*Sternoptyx pseudobscura*	MYH6	OP150088	RIE_537
*Sternoptyx pseudobscura*	MYH6	OP150089	RIE_538
*Sternoptyx pseudobscura*	MYH6	OP150090	RIE_538
*Sternoptyx pseudobscura*	MYH6	OP150091	RIE_539
*Sternoptyx pseudobscura*	MYH6	OP150092	RIE_539
*Sternoptyx pseudobscura*	MYH6	OP150093	RIE_78
*Sternoptyx pseudobscura*	MYH6	OP150094	RIE_78
*Stomias affinis*	MYH6	OP150095	DPND_1201
*Stomias affinis*	MYH6	OP150096	DPND_1201
*Stomias affinis*	MYH6	OP150097	DPND_1302
*Stomias affinis*	MYH6	OP150098	DPND_1302
*Stomias affinis*	MYH6	OP150099	DPND_1314
*Stomias affinis*	MYH6	OP150100	DPND_1314
*Stomias affinis*	MYH6	OP150101	DPND_1315
*Stomias affinis*	MYH6	OP150102	DPND_1315
*Stomias affinis*	MYH6	OP150103	DPND_1408
*Stomias affinis*	MYH6	OP150104	DPND_1408
*Stomias affinis*	MYH6	OP150105	DPND_1543
*Stomias affinis*	MYH6	OP150106	DPND_1543
*Stomias affinis*	MYH6	OP150107	DPND_1639
*Stomias affinis*	MYH6	OP150108	DPND_1639
*Stomias affinis*	MYH6	OP150109	DPND_3645
*Stomias affinis*	MYH6	OP150110	DPND_3645
*Stomias affinis*	MYH6	OP150111	RIE_466
*Stomias affinis*	MYH6	OP150112	RIE_466
*Stomias affinis*	MYH6	OP150113	RIE_577
*Stomias affinis*	MYH6	OP150114	RIE_577
*Stomias affinis*	MYH6	OP150115	RIE_917
*Stomias affinis*	MYH6	OP150116	RIE_917

**TABLE A2:** List of primers used.

Gene	Primer name	Primer sequence
COI	FISH1F	TCAACCAACCACAAAGACATTGGCAC
COI	FISH1R	TAGACTTCTGGGTGGCCAAAGAATCA
COI	FISH2F	TCGACTAATCATAAAGATATCGGCAC
COI	FISH2R	ACTTCAGGGTGACCGAAGAATCAGAA
COI	BOLD_COI_Forward	TTCTCCACCAACCACAARGAYATYGG
COI	BOLD_COI_Reverse	CACCTCAGGGTGTCCGAARAAYCARAA
COI	FISHCOI_F	TCAACYAATCAYAAAGATATYGGCAC
COI	FISHCOI_R	ACTTCYGGGTGRCCRAARAATCA
ENC	Perc_ENC_F	TTCCTRGAGAGAAACCTTCACC
ENC	Perc_ENC_R	GAYGGAGARGCNGGGAGGCAGCC
PLAG	Perc_PLAG_F	CATGAYCCYAACAARGARGCCTT
PLAG	Perc_PLAG_R	TGRCARCCCATGCCCATAGCTG
MYH	Perc_MYH_F	ACYAARAGRGTYATYCAGTACT
MYH	Perc_MYH_R	CCRAKGGMRTAGTAGACYTGRTC

**TABLE A3:** Range description of study species.

Species	Range description	Oceans inhabited	Latitudes inhabited	Citations	Notes
*Bathophilus pawneei*	Circumglobal; Tropical	Atlantic, Indian, Pacific	36° N–34° S	Agustin (2018)	
*Chauliodus sloani*	Circumglobal; Tropical and Polar	Atlantic, Indian, Pacific	50° N–50° S	Mundy (2005)	Less common but records exist from individuals as far as 70° N–56° S (Priede, 2017)
*Cyclothone alba*	Circumglobal; Tropical	Atlantic, Indian, Pacific	40° N–40° S	Miya and Nemoto (1986)	
*Cyclothone pseudopallida*	Circumglobal; Tropical and Polar	Atlantic, Indian, Pacific	65° N–30° S	Mundy (2005)	
*Diplospinus multistriatus*	Circumglobal; Tropical	Atlantic, Indian, Pacific	40° N–40° S	Mundy (2005)	
*Ditropichthys storeri*	Circumglobal; Tropical	Atlantic, Indian, Pacific	48° N–43° S	Paxton (1989)	
*Photostomias guernei*	Non circumglobal; Tropical	Atlantic	40° N–3° N	Kenaley (2009)	
*Scopelogadus mizolepis*	Circumglobal; Tropical	Atlantic, Indian, Pacific	40° N–22° S	Mundy (2005)	
*Sigmops elongatus*	Circumglobal; Tropical and Polar	Atlantic, Indian, Pacific	65° N–35° S	Torres (2018)	
*Sternoptyx pseudobscura*	Circumglobal; Tropical	Atlantic, Indian, Pacific	40° N–40° S	Mundy (2005) and Zamarro and Lloris (1999)	One record from 42° N and 47° N
*Stomias affinis*	Circumglobal; Tropical	Atlantic, Indian, Pacific	35° N–39° S	Priede (2017)	
